# The Influence of External Additional Loading on the Muscle Activity and Ground Reaction Forces during Gait

**DOI:** 10.1155/2021/5532012

**Published:** 2021-07-29

**Authors:** Bartłomiej Zagrodny, Michał Ludwicki, Wiktoria Wojnicz

**Affiliations:** ^1^Department of Automation, Biomechanics and Mechatronics, Faculty of Mechanical Engineering, Łódź University of Technology, 1/15 Stefanowskiego Str. 90-924 Łódź, Poland; ^2^Faculty of Mechanical Engineering and Ship Technology, Gdansk University of Technology, 11/12 Narutowicza Str. 80-233 Gdańsk, Poland

## Abstract

Asymmetrical external loading acting on the musculoskeletal system is generally considered unhealthy. Despite this knowledge, carrying loads in an asymmetrical manner like carrying on one shoulder, with one hand, or on the strap across the torso is a common practice. This study is aimed at presenting the effects of the mentioned load carrying methods on muscle activity assessed by using thermal field and ground reaction forces. Infrared thermography and pedobarographic force platform (ground reaction force/pressure measurement) were used in this study. Experimental results point out an increased load-dependent asymmetry of temperature distribution on the chosen areas of torso and the influence of external loading on ground reaction forces. Results point out that wearing an asymmetrical load should be avoided and are showing which type of carrying the external load is potentially less and the most harmful.

## 1. Introduction

In a human body, there may exist physiological asymmetries in the musculoskeletal system at the level that we assume still in range of norm or already pathological. They can be understood as faulty posture (like scoliosis and different leg lengths) or connected with the kinetics of movement (when speaking about asymmetrical gait—different step lengths or ground reaction forces). Generally, almost any asymmetry in the musculoskeletal system is seen as a defect. One has to know that the problem can be increased by loading a body with asymmetrical, external loading. This can have a short- or long-term negative effect on posture correctness and/or static and dynamical stability. It can cause an injury or degeneration of muscles or joints. The consequence of that can be joints' kinematic and kinetic imbalance and body segments or components of the musculoskeletal system, either active and passive tissues, leading to an injury [1]. The muscular system of a healthy human undergoing symmetrical external loading should produce relatively symmetrical ground reaction forces (GRFs) during normal gait. It means that in nonpathological cases, the ground reaction forces for the left and right lower limb should be similar. Lack of GRF symmetry for both lower limbs can be linked with muscle imbalance or some problems with the nervous and/or musculoskeletal system (like joint degeneration, injuries [2], or asymmetrical body load [3] caused by external factors). Furthermore, the muscle activity should be symmetrical in the case of symmetrical loading. In contrary, asymmetrical loading causes asymmetrical muscle activation to compensate for the influence of asymmetry [4].

Muscle contraction leads to increased blood flow to supply the activated muscles with all necessary nutrients and oxygen as well as to remove metabolites [5, 6] and to cool the muscles down. The energy produced by muscles is mainly dissipated in the form of heat (up to 70% [7]) and is caused by its relatively low mechanical efficiency. In the thermal regulation process, one of the important elements is the skin. It plays an important role, and due to vasoconstriction, sweating, and shivering [8, 9], the human body can regulate the temperature of the body shell and core. By observing the skin temperature, the most and less active surface skeletal muscles can be selected. Some works point out that with the use of the infrared technique (IRT), the level of muscle activity in sport and during daily activity can be estimated [10, 11]. To do this, mainly an infrared camera is the most common choice [12–16]. It is possible to assess muscle activity even at a low level of their activity [17]. In the literature, one can find that temperature differences below 1°C are also considered, and when the experiments are carried out under controlled conditions, the thermal results are treated as scientifically significant [15, 18]. The thermal imaging technique proves also its usability in detecting asymmetrical muscle activity. In work [19], the influence of additional loads on chosen gait parameters and muscle activity was done with the meaning of thermal imaging and optoelectronic system.

In contrary to another popular method—surface electromyography (sEMG), which allows determining the level of muscle activation by the meaning of electrical signals [20, 21], IRT is a noncontact technique and allows observing the whole body, not the muscles chosen *a priori.*

Recording of ground reaction forces (GRFs) is widely used to examine a normal and pathological gait [22–24].

The influence of different types of additional external loading on muscle activation has been determined in numerous studies. Load in a form of a hockey bag of different sizes [25] or a backpack worn in different positions [26–29] was examined. The influence of carrying an additional load in one or both hands in the range from 5 up to 30 kg on muscle activation was investigated in work [30]. In all aforementioned papers, muscle activity was assessed by sEMG.

A bag is currently a common way of carrying the load. People keep them in hand, or hanging on the strap, put on the shoulders as support, or put a long strap across their torso. Carrying backpacks on one shoulder is also popular among the young generation [31]. These four methods of carrying additional loads have been examined.

This study is aimed at determining the relationship between different types of external asymmetrical musculoskeletal loading (backpack on one shoulder, bag in one hand, bag on one shoulder, and bag with the strap across the torso, with an additional linear distributed load normalized to the body weight of 5%, 10%, and 15%) and asymmetry of muscle activity assessed by using thermal fields of the torso chosen areas (*trapezius*, *latissimus dorsi*, and *obliquus abdominis*). That asymmetrical external load influences human posture and can be treated as a preliminary study as limited to young male volunteers. The additional loading is treated as an external perturbation. According to [32], it is important to gain a broader knowledge in the field of muscle coordination in daily life, especially when the musculoskeletal system undergoes different types of perturbations.

## 2. Materials and Methods

Infrared thermography was used to assess torso muscle activity. The muscles chosen for analysis are right and left *latissimus dorsi*, right and left *trapezius*, and right and left *obliquus abdominis*. They were selected as the biggest and most significant muscles involved in maintaining the correct posture. An InfReC R300SR-S thermal camera (NEC-Avio, Japan, FPA-type sensor, spectral range 8−14·10−6 m and NETD 0.08 K) was used. The supplementary data were obtained from a pedobarographic force platform 1.5 m long with an additional 6 m walkway (Footscan, RSscan International, 12288 sensors in a 192 × 64 matrix, frequency up to 200 Hz). Additionally, a motorised treadmill (York Fitness) was employed in this study.

Experiments were done in monitored conditions, according to the protocol described in detail in [17]. All objects with high reflectance or temperature were removed from the surrounding. The ambient laboratory temperature could be chosen by the volunteer prior to the experiment in the range of 21°C–24°C. The humidity was in the range of 30%–45% RH (depending on the external conditions). No humidifiers or air-dryers were used. Both parameters were monitored during each experiment, stored, and used in further analysis. Air movement in the laboratory was minimised. Each participant had 20 minutes of thermal adaptation. The skin emissivity was set to 0.98. In each case, the skin was free of tattoo, inflammation, or other types of dermatological or vascular problems. To improve the reliability of the experiments, it was decided to ask volunteers to fulfil all additional restrictions described in [17]. The protocol is presented in detail in Supplementary Materials in Tables S1 and S2. The inclusion criteria for volunteers were as follows: male, age 20–27 years, and body core temperature below 37°C. The exclusion criteria were as follows: diagnosed neurological problems, cardiovascular drug treatment, leg length difference greater than 0.5 cm, failure to comply with the preparation rules of thermal imaging examination, skin inflammation, and visible “hot spots” on the body in IR, or failure to pass the restricted Romberg test.

Nine healthy male university students volunteered in this experiment. All were without any injuries, neuromusculoskeletal disorders, and visible asymmetry/faulty posture. To check for scoliosis, the Addams manoeuvre was used. Their age was in the range 23.5 ± 2.5; height, 181.1 ± 6.5 cm; body weight, 78.0 ± 18.5 kg; and body mass index, 23.7 ± 4.2 kg/m^2^. All participants declared as right hand and right leg dominant. Volunteers were instructed to walk barefoot during all trials. The experiment was organized according to the Helsinki regulation, and all participants were informed in detail about its aim, scope, and procedure and signed the written consent, accepted by the local ethical board (Committee of Research Ethics with Human Participation at Gdansk University of Technology).

**Figure 1:**
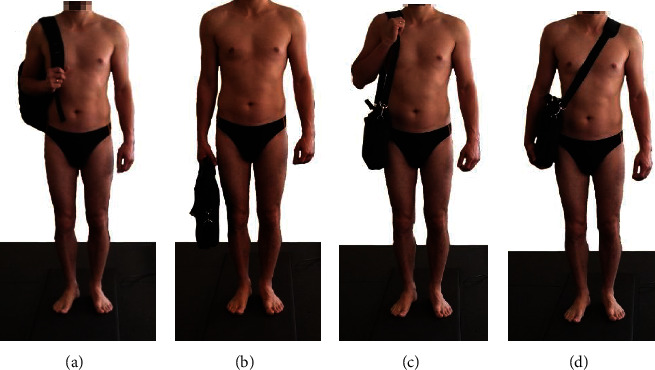
Four investigated methods of carrying the load: (a) backpack on one shoulder, (b) bag in one hand, (c) bag on one shoulder, and (d) bag with strap across torso.

Each participant was assigned randomly to carry the additional load in one of four different ways, as shown in Figure 1, i.e., respectively: (a) backpack on one shoulder (backpack), (2) bag in one hand (bag one hand), (3) bag on one shoulder (bag shoulder), and (4) bag with the strap across the torso (bag across), with an additional linear distributed load normalized to their body weight of 5%, 10%, and 15% (as in work [3]), as well as to perform a control gait without an additional load to determine the effects of each load.

The experimental procedure was identical for each participant and was as follows: firstly, volunteers were asked to remove clothing from the upper body and to acclimate for 20 minutes to obtain stable skin temperature. Next, initial upper-body thermograms were taken (*anterior* and *posterior* side of the torso) with the thermal camera positioned 3 m away from subjects on a tripod. Then, volunteers were asked to walk on a pedobarographic force platform with the same load and type of carrying as it was done for thermal imaging. Next, the main task starts (within 30 s) with a gait on a motorised treadmill for a 1 km distance with a velocity equal to 4 km/h. This speed was chosen as an average, comfortable speed for most of the volunteers after pretrials, which is slower by 0.5 km/h than the comfortable speed on a treadmill mentioned in [17] as a result of a natural tendency to walk slower when carrying a load [4]. A second thermogram was taken right after the gait sequence (also within 30 s; this time takes to move from the front of camera to the treadmill). A third thermal image was taken 5 minutes after the second one due to the presence of sweat on the skin after the activity (especially in places where the bag strap contacts the skin). It is worth noticing that due to thermoregulation and especially sweating which has a cooling effect on the skin [33], it was decided to examine the asymmetry of temperature distribution and changes of this asymmetry as an indicator of uneven loading of the left and right muscle part (difference: left − right).

Each sequence of mentioned measures (one type of carrying the load with given level) took approximately 45 minutes per person. The next weight/load type combination was done on a different day to minimise the influence of each set on another one.

**Figure 2:**
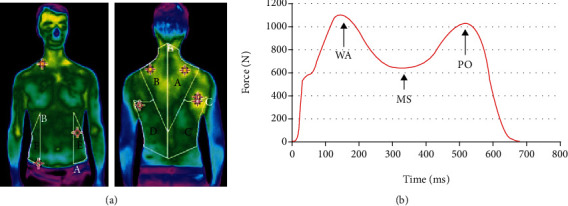
(a) Exemplary thermal image of a volunteer with muscles marked in the software: A/B—*trapezius* (right/left), C/D—*latissimus dorsi* (right/left), and E/F—*obliquus abdominis* (left/right). (b) Chosen results of ground reaction forces.

The results of thermal imaging were analysed in the dedicated software InfReC Analyzer NS9500 Standard. For each volunteer, the areas of the left and right *trapezius*, *latissimus dorsi*, and *obliquus abdominis* were marked as shown in Figure 2(a). Additionally, the whole trunk skin average temperature was measured just before (second thermogram) and 5 minutes after gait (third thermogram), separately for the *ventral* and *dorsal* part of the torso. The results presented as the change of average temperature were calculated as the difference of average temperatures between the right and the left muscle, before and after each experiment (first and second thermogram).

The results for the pedobarographic force platform were also analysed in dedicated software, later exported for further calculations. Volunteers performed 5 crossings on a pedobarographic force platform. For further analysis, automatically calculated average results of these 5 crossings were used. Walk on the pedobarographic force platform was performed just after finishing the first thermal imaging with the same weight/load type combination. Three ground reaction forces were considered (see Figure 2(b)): maximal weight acceptance force (WA), force in midstance (MS) (local minimum), and maximal force in push-off gait phase (PO).

Using the Shapiro-Wilk test, a normality of data distribution was verified. To set linear relationships for normal distributed groups, the Pearson correlation coefficient *r* was defined by considering the statistical significance threshold *p* = 0.1. To define linear relationships for nonnormal distributed groups, the Spearman correlation coefficient *r*^∗^ was used by assuming the statistical significance threshold *p* = 0.05.

Linear regressions were set between three measured muscle group temperature mean differences (left side minus right side) and three measured ground reaction forces (WA, MS and PO). The statistical calculations were performed by using the StatSoft Statistica 13.1 package. Trying to classify the strength of the correlation relationship, we adopted the following ranges, given in [34]: (0; 0.2]—poor, (0.2; 0.5]—fair, (0.5; 0.7]—moderate, (0.7; 0.9]—very strong, and (0.9; 1.0]—perfect.

## 3. Results

### 3.1. Thermal Imaging

**Figure 3:**
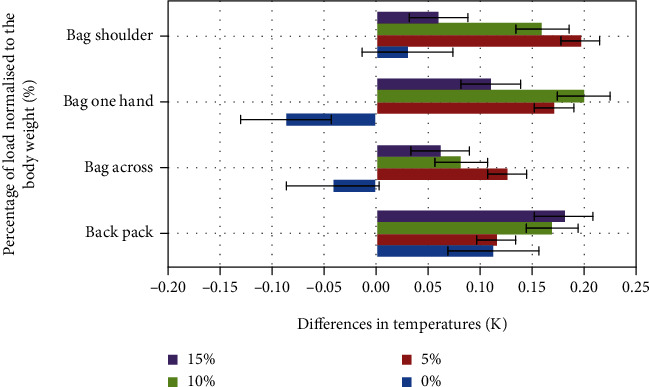
Average differences for *latissimus* in the function of normalized load.

**Figure 4:**
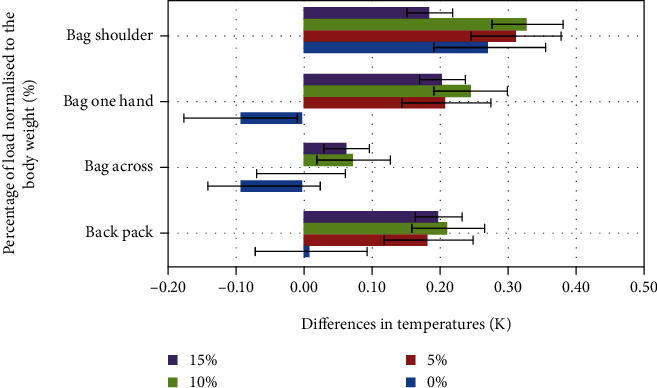
Average differences for *trapezius* in the function of normalized load.

**Figure 5:**
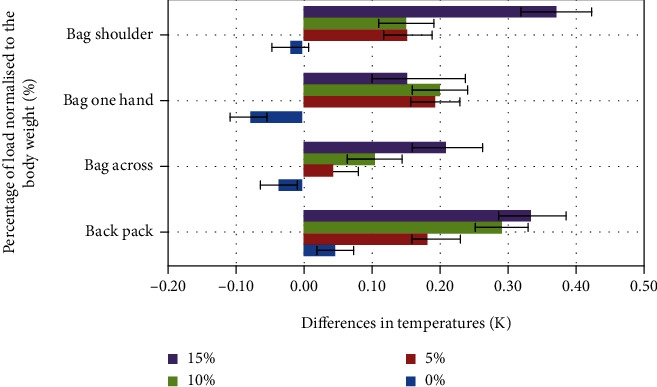
Average differences for *obliquus abdominis* in the function of normalized load.

Results of thermal measurements were assessed as average values with standard deviations for all volunteers (Figures 3–5). They should be interpreted as an asymmetry of temperature distribution on the chosen muscle area (*trapezius*—right/left, *latissimus dorsi*—right/left, and *obliquus abdominis*—left/right) after the exercise. In each case, the reference level was a thermal image done just before the experiment, after acclimatization. The presented values are relative and calculated as difference: left (L) − right (R).

The initial asymmetry in temperature distribution varies from 0.04 K for the *obliquus abdominis* and 0.07 K for the *latissimus dorsi* up to 0.11 K for the *trapezius* muscle. The highest differences after the experiment were reached for the *obliquus abdominis* (Figure 5) 15% load carried on the shoulder (0.37 K). For the *latissimus dorsi* (Figure 3), the highest asymmetry was observed in the case of 10% load, bag in one hand (0.2 K), and slightly less for bag on one shoulder with 5% of the load (0.19 K). For the *trapezius* (Figure 4) muscle, the highest asymmetry was observed for the bag held on one shoulder with 10% of body load (10% increase).

In the case of the *latissimus dorsi* and *obliquus abdominis* with additional external loading on the right side, the left side of the muscles was warmer in comparison to the right side. In the case of the *trapezius* muscle, we can observe an opposite phenomenon, and this can be explained by the scapula and clavicle stabilization done by this muscle.

### 3.2. Pedobarographic Examination

**Figure 6:**
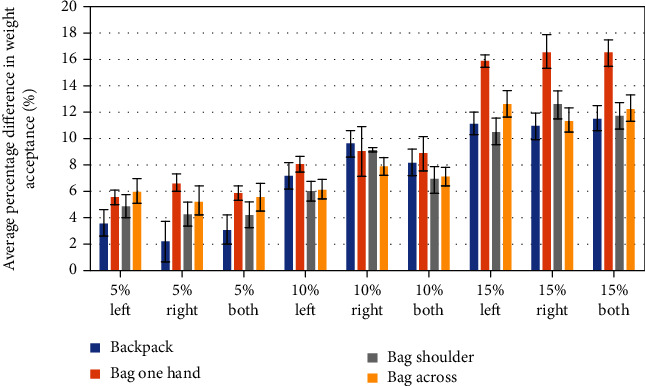
Maximal weight acceptance changes for the left, right, and average of both (left and right side) for different levels of additional load.

**Figure 7:**
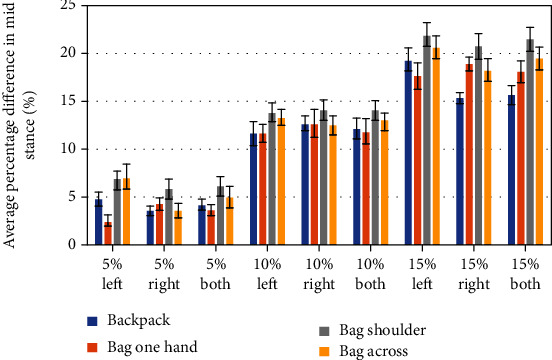
Midstance force changes for the left, right, and average of both (left and right side) for different levels of additional load.

**Figure 8:**
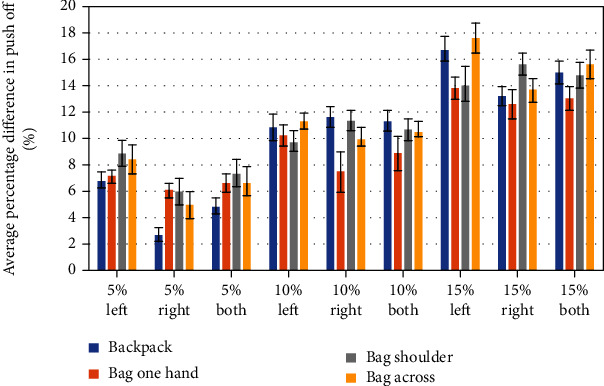
Push-off forces for the left, right, and average of both (left and right side) for different levels of additional load.

Figures 6–8 are presenting the percentage ratio of maximal weight acceptance force (WA) (Figure 6), maximal force in midstance (MS) (Figure 7), and maximal force in push-off (PO) (Figure 8) as averages for all volunteers with standard deviations. The value “both” means an average for the left and right site.

Results for all types of load are presented regarding each time to the nonloaded case. As it can be observed, an increase in WA, MS, and PO forces is visible in all cases of external loading. The WA was the highest in the case when the bag was carried in one hand. This result can indicate the impact of this type of carrying on the gait dynamics and its stability [35].

The highest values for MS are obtained for the bag carried on one shoulder, and the lowest are surprisingly for the bag carried in one hand. The hypothesis is put forward; it relates to balance in the frontal plane, but it needs deeper investigation. In the case of PO force, it cannot be distinguished by any dominant type of load/carrying method that generates the highest values; thus, only a graduation from the lowest to highest values is seen, dependent on the value of external loading. There is no statistically significant difference for the majority of cases between the forces recorded for the left or right leg; similar conclusions are published in paper [12].

### 3.3. Relationship Investigation

According to the tests performed, it was defined that 104/108 samples related to thermal parameters and force parameters have normal distributions. In Table 1 are given statistically significant results for one side (right or left) or both sides between thermal parameters (*trapezius* (right/left), *latissimus dorsi* (right/left), and *obliquus abdominis* (left/right)) and force parameters (maximal weight acceptance force (WA), maximal force in midstance (MS), and maximal force in push-off gait phase (PO)). For normal distributed sets, the Pearson coefficients and coefficient of determination *r*^2^ are given (*p* = 0.1). For nonnormal distributed sets, the Spearman coefficients are presented (*p* = 0.05).

## 4. Discussion

The experiment was focused on the effects of an asymmetrical load on the work of trunk muscles and differences in ground reaction forces. In particular, the asymmetry of temperature distribution was observed (Figures 3–5). The highest differences were reached for the *obliquus abdominis* in the case of 15% load carried on the shoulder (0.37 K) and for the *latissimus dorsi* for 10% load in the case of a bag carried in one hand (0.2 K), and it was slightly less for a bag on one shoulder with 5% of the load (0.19 K). For the *trapezius* muscle, the highest asymmetry was reached for the bag carried on one shoulder with 10% of body load (10% increase).

Based on those results, we can assume that a COM (center of mass) translation and compensation of the asymmetrical load cause a counterbalance of spine lateral flexion and lead to asymmetrical trunk muscle activation.

As expected, almost any asymmetrical load induces an increase in the asymmetry of temperature distribution, and generally, it can be stated that higher asymmetrical load causes higher asymmetry in temperature distribution. The main exception to the rule is the case of the highest load—15% of the body mass. It can be assumed that the additional load influences the position of the center of mass which changes the kinematics of the body and excessive physical effort causes sweating, and this influences the temperature distribution. Similar phenomena were observed in other works [36, 37].

Results reveal asymmetrical muscle work caused by their asymmetric activity and force production caused by an asymmetric external load. A similar testing procedure to the presented one was done in work [38], but the technique of muscle activity recording was surface electromyography. Results in the mentioned study pointed out statistically significant differences only for the *trapezius* and *erector spinae* but not statistically significant differences in *latissimus dorsi* and *obliquus abdominis* activity.

Results presented in this study showed such dependence in all cases except for the cross-body bag. However, one might try to define nonlinear relationships between thermal and ground reaction force parameters, but this demands to test a bigger number of subjects. According to the experiment carried out and presented in the paper [39], the biggest differences should be visible for the volunteer carrying a bag in the position lower than the level of the shoulder. In this study, this is not proven. For most volunteers, it may be concluded that the posture is not exactly symmetrical according to the sagittal plane and this is perfectly normal.

Additional COM translation verification may be considered here, e.g., by using motion capture or IMU systems [40].

The results of the experiment carried out with children's participation [41] prove the asymmetrical muscle activity among those with the problem of scoliosis with one curve as well as double curve one. In the presented study, the temperature difference between right and left muscles before any activity is positive value and shows an asymmetrical muscle activity. Generally, the *trapezius* muscle is increasingly activated with increasing external load on the right side. If the load is not distributed bilaterally, there is an increased muscle activity of the superior part of the *trapezius* on the side that the bag is worn on. The same conclusion was found in the publication [38]. In all examined cases where the additional load was distributed nonuniformly, the *trapezius* was more activated on the side where the strap was held on. This is probably due to the volunteers trying to maintain the proper *scapula* and *clavicle* position to ensure the strap of the bag is kept over the shoulder while the trunk is laterally flexed so that the center of mass of the body remains over the support area during gait.

The asymmetry of GRFs is revealed in Figures 6–8. Generally, the highest asymmetry is obtained for MS forces. Based on the obtained results and those found in literature, we can assume that a COM translation and compensation of the asymmetrical load present in a form of counterbalance cause spine lateral flexion and result in asymmetrical trunk muscle activation. Repeating this type of asymmetrical loading of the musculoskeletal system is linked with greater shear and compressive forces present in the spine [37]. The authors of [37] pointed out also that “asymmetric lifting is more stressful than frontally symmetric lifting.”

Considering the backpack load type (carried on the right side of the body), increased activation of the right *abdominis* and right *latissimus* muscles should be expected. The bigger number of statistically significant linear correlations was obtained for the right side, mostly for the *trapezius* and *latissimus dorsi* muscles (see Table 1).

Considering a bag carried in the right hand, increased activation of the right opposite *trapezius*, *latissimus*, and *abdominis* should be expected. The position of external load with respect to the COM of the body is the most distant with respect to the other configurations of the load, and that is why one should expect that this carrying could be the most fatigable ones. To avoid fatigue, the musculoskeletal should activate different muscle groups, and that is why the lowest number of statistically significant linear correlation (for *trapezius* and *latissimus dorsi* muscles) was found in this case (see Table 1).

Considering a bag carried on right shoulder, increased activation of the right *trapezius*, *abdominis*, and *latissimus* should be expected. The number of statistically significant linear correlations is similar to those identified for the bag carried in one hand but mostly for the left side of the body and for the *obliquus abdominis* muscle (see Table 1).

Considering a bag carried across the body (on the right side) (the best one), one can induce that external load is stabilized, and that is why this is the less fatigable position. Increased activation of contraction of the opposite *trapezius* and *abdominis* should be expected.

The bigger number of statistically significant linear correlations was obtained for the left side and only for *obliquus abdominis* and *latissimus dorsi* muscles (see Table 1).

## 5. Conclusions

Obtained results show that during gait with an additional load held asymmetrically, the symmetry of muscle force production changes. With the increasing weight of the carried load, the differences of temperature become higher; however, different types of loading cause different patterns of compensation and influence the ground reaction forces in different ways.

A general observation allows us to make a statement. that in the case of the asymmetrical way of carrying the external load, the less harmful for the musculoskeletal system seems to be the placing the strap across the torso because in this case, the smallest increase in temperature asymmetry was observed. On the other hand, the worst method is to keep the load in one hand or on the shoulder especially when we use to carry the load only one side almost every time—in this case, the highest increase in temperature asymmetry was present.

Generally, it should be underlined that the results indicate that walking with the asymmetric load inducts compensation made by muscles and posture and increases the possibility of muscle injury and leads to or increases faulty posture, even if this study will be treated as a preliminary study, limited to relatively young and fit males.

## Figures and Tables

**Table 1:** Statistical relationships between thermal and force parameters for three muscle groups: *trapezius* (TT), *latissimus dorsi* (TL), and *obliquus abdominis* (TO), and three ground reaction force values: maximal weight acceptance force (WA), maximal force in midstance (MS), and maximal force in push-off gait phase (PO), for the left and right leg, respectively. Significant relations (*p* < 0.1 or *p* < 0.05 for Spearman's test) are written in bold.

Load (%)	Load type	Force	Muscle	Left side				Right side			
				*r* or *r*^∗^	*p*	*r* description	*r* ^2^	*r* or *r*^∗^	*p*	*r* description	*r* ^2^
5%	Backpack	MS	TL	-0.757	**0.018**	Very strong negative	0.573	0.311	0.415	Fair positive	0.097
5%	Backpack	MS	TT	-0.233	0.547	Fair negative	0.054	0.878	**0.002**	Very strong positive	0.771
5%	Backpack	PO	TL	-0.852	**0.004**	Very strong negative	0.726	0.881	**0.002**	Very strong positive	0.777
5%	Backpack	WA	TO	0.691	**0.039**	Moderate positive	0.477	0.123	0.753	Poor positive	0.015
10%	Backpack	PO	TL	0.351	0.354	Fair positive	0.123	-0.629	**0.069**	Moderate negative	0.396
10%	Backpack	WA	TL	0.177	0.649	Poor positive	0.031	-0.805	**0.009**	Very strong negative	0.649
15%	Backpack	MS	TT	0.134	0.731	Poor positive	0.018	-0.867	**0.002**	Very strong negative	0.752
15%	Backpack	WA	TT	0.135	0.729	Poor positive	0.018	-0.621	**0.075**	Moderate negative	0.385
15%	Bag across	MS	TO	-0.707	**0.033**	Very strong negative	0.500	0.586	**0.097**	Moderate positive	0.344
15%	Bag across	PO	TL	-0.696	**0.037**	Moderate negative	0.485	0.130	0.739	Poor positive	0.017
15%	Bag across	WA	TL	-0.382	0.311	Fair negative	0.146	0.733	**0.025**	Very strong positive	0.537
15%	Bag across	WA	TO	-0.334	0.380	Fair negative	0.111	0.711	**0.032**	Very strong positive	0.505
5%	Bag one hand	PO	TT	0.378	0.209	Fair positive	0.143	-0.585	**0.098**	Moderate negative	0.342
15%	Bag one hand	MS	TL	-0.756	**<0.05**	Very strong negative	—	0.235	>0.05	Fair positive	—
15%	Bag one hand	MS	TT	0.031	0.937	Poor positive	0.001	-0.599	**0.088**	Moderate negative	0.359
15%	Bag one hand	PO	TL	-0.807	**<0.05**	Very strong negative	—	0.521	>0.05	Moderate positive	—
15%	Bag one hand	WA	TL	-0.277	>0.05	Fair negative	—	0.731	**<0.05**	Very strong positive	—
15%	Bag one hand	WA	TT	0.636	**0.066**	Moderate positive	0.404	-0.026	0.947	Poor negative	0.001
5%	Bag shoulder	MS	TO	0.792	**0.011**	Very strong positive	0.628	-0.385	0.306	Fair negative	0.148
15%	Bag shoulder	MS	TL	0.586	**0.097**	Moderate positive	0.343	-0.240	0.534	Fair negative	0.058
15%	Bag shoulder	MS	TO	0.329	0.387	Fair positive	0.108	-0.641	**0.063**	Moderate negative	0.411
15%	Bag shoulder	PO	TO	0.673	**0.047**	Moderate positive	0.453	-0.259	0.501	Fair negative	0.067
15%	Bag shoulder	PO	TT	-0.373	0.322	Fair negative	0.139	0.750	**0.020**	Very strong positive	0.562
15%	Bag shoulder	WA	TO	0.757	**0.018**	Very strong positive	0.573	-0.132	0.736	Poor negative	0.017

## Data Availability

The thermography image data and pedobarographic force platform data used to support the findings of this study are available from the corresponding author upon request.

## Supplementary Materials

Supplementary Materials Table S1: protocol for thermal imaging examination with a checklist used in the laboratory. Table S2: laboratory conditions with a checklist.
