# Supplementary material for: The Influence of External Additional Loading on the Muscle Activity and Ground Reaction Forces during Gait
Source: Appl Bionics Biomech. 2021 Jul 29;2021:5532012. doi: 10.1155/2021/5532012 (PMC8342170; doi:10.1155/2021/5532012)
Supplement: Supplementary Materials — Table S1: protocol for thermal imaging examination with a checklist used in the laboratory. Table S2: laboratory conditions with a checklist. [file 5532012.f1.docx]

# Applied Bionics and Biomechanics

# The influence of external additional loading on the muscle activity and ground reaction forces during gait

Bartłomiej Zagrodny,^1^ Michał Ludwicki,^1^ Wiktoria Wojnicz^2^

^1^ Department of Automation, Biomechanics and Mechatronics, Faculty of Mechanical Engineering, Łódź University of Technology, 90-924, Stefanowskiego Str. 1/15, Łódź, Poland
^2^ Faculty of Mechanical Engineering and Ship Technology, Gdansk University of Technology, Narutowicza 11/12 str. Gdańsk, Poland

## Supplementary Materials

Table S1. Protocol for thermal imaging examination with a check list used in the laboratory.

| No. | Requirement | Minimum before examination  (d-days,  h-hours) | Fulfilled (YES) | Not Fulfilled (NO) | Remarks |
| --- | --- | --- | --- | --- | --- |
| 1. | No sunbathing | 5d |  |  |  |
| 2. | Not to use a cream, lotion, perfume, body-deodorant or antiperspirant or other types of on-skin preparations | 2d |  |  |  |
| 3. | To avoid physical activity (especially exercises), massage, electrostimulation, ultrasonography, acupuncture, hot or cold compress | 2d |  |  |  |
| 4. | If it is necessary – to remove body-hair (from examined area by short-trimming it) | 24h |  |  |  |
| 5. | Do not drink any amount of alcohol | 24h |  |  |  |
| 6. | To do last bath (best short shower) in lukewarm water (around 36^◦^C) without soap or other type of detergent. It is advised to bath like this during 3 days before examination | 12h |  |  |  |
| 7. | Do not use any tight-fitting garment parts | 12h |  |  |  |
| 8. | To avoid any kind of excessive activity (run, fast gait, climbing stairs etc.) | 4h |  |  |  |
| 9. | Do not drink coffee or strong tee nor energy drink | 4h |  |  |  |
| 10. | Do not eat or drink hot/cold drinks | 2h |  |  |  |
| 11. | Do not smoke and/or use nicotine | 2h |  |  |  |
| 12. | To inform the investigator about vasoactive drugs used last time |  |  |  |  |

Table S2. Laboratory conditions with a checklist.

| No. | Requirement | Fulfilled (YES) | Not Fulfilled (NO) | Remarks |
| --- | --- | --- | --- | --- |
| 1. | Size of the lab: minimum 4x4x2.5m |  |  |  |
| 2. | Temperature in the laboratory in range 21-24◦C, stabilized during experiment up to 0.5◦C |  |  |  |
| 3. | Humidity around 45% RH Stabilized during experiment |  |  |  |
| 4. | Advection and convection reduced to minimum |  |  |  |
| 5. | All IR radiators (with temperature higher than 20◦C than surrounding – removed from the lab, if impossible - covered |  |  |  |
